# Type III Collagen is Required for Adipogenesis and Actin Stress Fibre Formation in 3T3-L1 Preadipocytes

**DOI:** 10.3390/biom11020156

**Published:** 2021-01-25

**Authors:** Mohammad Al Hasan, Patricia E. Martin, Xinhua Shu, Steven Patterson, Chris Bartholomew

**Affiliations:** 1 Department of Biological & Biomedical Sciences, School of Health & Life Sciences, City Campus, Glasgow Caledonian University, Cowcaddens Road, Glasgow G4 OBA, UK; Mohammad.al.hasan@outlook.com (M.A.H.); patricia.martin@gcu.ac.uk (P.E.M.); xinhua.shu@gcu.ac.uk (X.S.); steven.patterson@gcu.ac.uk (S.P.); 2 Department of Vision Science, School of Health & Life Sciences, City Campus, Glasgow Caledonian University, Cowcaddens Road, Glasgow G4 OBA, UK

**Keywords:** type III collagen, *COL*3A1, adipogenesis, genome editing, extracellular matrix, actin cytoskeleton

## Abstract

GPR56 is required for the adipogenesis of preadipocytes, and the role of one of its ligands, type III collagen (ColIII), was investigated here. ColIII expression was examined by reverse transcription quantitative polymerase chain reaction, immunoblotting and immunostaining, and its function investigated by knockdown and genome editing in 3T3-L1 cells. Adipogenesis was assessed by oil red O staining of neutral cell lipids and production of established marker and regulator proteins. siRNA-mediated knockdown significantly reduced *Col*3a1 transcripts, ColIII protein and lipid accumulation in 3T3-L1 differentiating cells. *Col*3a1^−/−^ 3T3-L1 genome-edited cell lines abolished adipogenesis, demonstrated by a dramatic reduction in adipogenic moderators: Pparγ_2_ (88%) and C/ebpα (96%) as well as markers aP2 (93%) and oil red O staining (80%). *Col*3a1^−/−^ 3T3-L1 cells displayed reduced cell adhesion, sustained active β-catenin and deregulation of fibronectin (*Fn*) and collagen (*Col*4a1, *Col*6a1) extracellular matrix gene transcripts. *Col*3a1^−/−^ 3T3-L1 cells also had dramatically reduced actin stress fibres. We conclude that ColIII is required for 3T3-L1 preadipocyte adipogenesis as well as the formation of actin stress fibres. The phenotype of *Col*3a1^−/−^ 3T3-L1 cells is very similar to that of *Gpr*56^−/−^ 3T3-L1 cells, suggesting a functional relationship between ColIII and Gpr56 in preadipocytes.

## 1. Introduction

Recent studies have shown that the G protein-coupled receptor 56 (GPR56 or ADGRG1) is required for adipogenesis. *Gpr*56 (*AdgrgI*) gene knockdown (KD) and genome editing in 3T3-L1 cells (*Gpr*56^−/−^) inhibit adipogenesis [1]. *Gpr*56^−/−^ 3T3-L1 cells show reduced proliferation and adhesion, changes in expression of extracellular matrix (ECM) genes, loss of actin stress fibres and sustained levels of active β-catenin, all of which may contribute to the inhibition of the adipocyte differentiation programme observed [1].

Type III collagen (COLlIII) binds GPR56 to regulate a range of biological activities, including brain development [2] and pancreatic islet β-cell function [3]. The interaction of COLIII with GPR56 stimulates RHOA activation via G-proteins Gα12/Gα13 [2]. The association of these two molecules raises the possibility that ColIII, like Gpr56, might also regulate adipogenesis.

Animal models show that the aberrant accumulation of ECM components has a negative impact on adipose tissue [4]. Collagens play an important role in the development of adipose tissue. They are major structural components of the ECM with roles in numerous biological processes, including ECM organisation, cell proliferation, adhesion and differentiation [5]. Preadipocyte differentiation to adipocytes is accompanied by ECM remodelling of collagen types I, III and V to collagen types IV and VI [6]. Transforming growth factor β inhibits 3T3-L1 adipogenesis and is accompanied by changes in collagens [7]. Chemical-mediated inhibition of collagen biosynthesis also inhibits adipocyte differentiation [8].

Changes in ColIII production normally accompanies adipogenesis, but a possible role in this differentiation process has not been investigated before. Although its function in adipocyte development is not fully elucidated, recent studies suggest a role. Protein–protein interaction networks show that it interacts with several proteins involved in regulatory cascades of adipogenesis [9]. Type I and III collagens are expressed in adipose tissue where they form collagen fibrils that support structure [10]. ColIII is reduced during 3T3-L1 cell adipocyte differentiation, which suggests a role in the regulation of adipogenesis [11]. ColIII is encoded by the *Col*3a1 gene, which comprises 51 exons and is composed of α-chain polypeptides consisting of repeating glycine–X–Y triplet amino acid units, where X and Y are frequently proline and hydroxyproline [12]. Three α-chain polypeptides form a homotrimer that associates with type I collagen to form collagen fibrils in adipose as well other tissues [5]. *Col*3a1-knockout (KO) mice (*Col*3a1^−/−^), which lack ColIII, are viable with a shortened lifespan and a similar connective tissue disorder phenotype to human vascular Ehlers–Danlos syndrome (vEDS) [13] and bilateral frontoparietal polymicrogyria [14,15].

ColIII is a key component of preadipocytes [16]. In this study, the role of ColIII in the differentiation of 3T3-L1 preadipocytes through the adipogenic developmental programme is investigated. The impact of both reduction and loss of ColIII upon differentiation is examined in *Col*3a1 knockdown (KD) and genome-edited cells, respectively. We show that ColIII is required for both adipogenesis and the formation of actin stress fibres in 3T3-L1 cells. Furthermore, we show that preadipocytes lacking ColIII are phenotypically similar to *Gpr*56^−/−^ 3T3-L1 cells, which lack the Gpr56 receptor for this ligand [1].

## 2. Materials and Methods

### 2.1. Cell Culture

3T3-L1 (ATCC®CL-173™) and RM4.2.5.5 (3T3-L1 *Gpr*56^−/−^) cells [1] were cultured in complete medium (CM), comprising Dulbecco’s Modified Eagle Medium (Lonza Group Ltd., Basel, Switzerland, BE12-604F), 10% *v*/*v* newborn calf serum (Merck, Gillingham, Dorset, UK, N4637), 2.5 mM glutamine, 50 units/ml penicillin and 50 µg/ml streptomycin (Lonza Group Ltd., BE17-605E and BE17-603E). Cell numbers were determined using a haemocytometer (Neubauer Marienfeld, GmbH & Co. KG, Lauda-Königshofen, Germany) and inverted microscope (CK2, Olympus UK Ltd., Southend-on-Sea, UK).

For differentiation, cells were kept confluent for 48 h prior to the addition of induction medium 1 (IM1 comprising CM with 10% *v*/*v* foetal calf serum Lonza, DE14-801F, 5 µg/mL insulin, 0.25 µM dexamethasone and 0.5 mM isobutylmethylxanthine, Merck, I9278, D4902 and I5879) for 48 h, followed by induction medium 2 (IM2 comprising CM with 10% foetal calf serum and 5 µg/ml insulin) every 48 h for up to 10 days.

Transfection of semi-confluent cells with 1 µg of each plasmid DNA (recombinant gRNA, hCas9 (gift from George Church, Addgene plasmid 41,824, 41,815 and pC1 [1]) for genome editing was performed with Lipofectamine 3000 according to the manufacturer’s instructions (ThermoFisher Scientific Inc., Sankt Leon-Rot, Germany, L300000). Transfected cells were transiently selected for 96 h with 2 µg/mL puromycin (P8833, Merck). Single cell clones were derived from isolated colonies using trypsin–EDTA (Lonza BE17-161E) saturated sterile filter paper disks (Whatmann 1MM, Fisher Scientific, Loughborough, UK) as described previously [1].

For cell adhesion assays, one thousand cells were seeded per well in 96-well plates and incubated for 6 h at 37 °C and 5% CO_2_. Cells were washed in PBS, fixed in 10% (*v*/*v*) formaldehyde/PBS for 10 min at rt then rinsed twice with PBS prior to staining for 10 min at rt in 0.1% (*w*/*v*) crystal violet (Merck C0775)/2% ethanol. Stained cells were washed in PBS and crystal violet extracted with 2% sodium dodecyl sulphate. Staining was quantified by measuring absorbance at 570 nm (plate reader, Epoch, Biotek Instruments Inc., Plainfield, NJ, USA).

Cells were washed in ice-cold PBS and fixed in 10% *v*/*v* formaldehyde for 10 min at rt in preparation for oil red O staining. Fixed cells were sequentially incubated for 1 h at rt in 6% *w*/*v* oil red O (Merck, O0625)/60% isopropanol followed by ice-cold PBS. Oil red O stain was extracted for quantification in a 50% culture volume of isopropanol for 5 min at rt. Absorbance was measured at 492 nm using an Epoch plate reader.

### 2.2. Preparation of Total Cellular RNA, cDNA Synthesis and Quantitative Real-Time Polymerase Chain Reaction

Total cellular RNA was prepared from cell cultures using TRI®Reagent according to the manufacturer’s instructions (Merck, 93,289). One microgram of total cellular RNA was used for cDNA synthesis with a SuperScript III First-Strand Synthesis Supermix cDNA synthesis kit (Invitrogen, 18,080), and 1.5% of the cDNA was used for quantitative real-time polymerase chain reaction with SYBR Green Rox mix (ThermoFisher, 11873913) and gene-specific oligonucleotide primers in a CFX96 C1000 thermal cycler (BIO-RAD Laboratories Ltd., Hemel Hempstead, UK). Analysis of relative expression levels between target and calibrator genes used the 2^−∆∆Ct^ method [17].

### 2.3. Western Blot Analysis

Total protein cell extracts were prepared in radioimmunoprecipitation assay buffer (RIPA buffer, 1% NP-40, 50 mM Tris pH 7.6, 120 mM NaCl, 1 mM EDTA) with protease and phosphatase inhibitors (Sigma-Aldrich P8340 and P0044), quantified using the Bicinchoninic acid protein assay method and analysed by SDS-PAGE as described previously [1]. Western blotting was performed with primary antibodies to PPARγ (C26H12), C/EBPα (2295), β−actin (8H10D10), active β-catenin (D13A1) (Cell Signalling Technologies ^®^, Leiden, The Netherlands), aP-2 (C-15), collagen type III alpha 1 (B-10 (Santa Cruz Biotechnology Inc) or collagen III (ab7778, Abcam PLC, Cambridge, UK) at 1:1000 dilution. IRDye® secondary antibodies (LI-COR™, Lincoln, NE, USA) for detection with an Odyssey FC image analyser (LI-COR®) was used and images were analysed with Image Studio Software Lite Ver 5.2 (LI-COR^®^). Quantitation was relative to nitrocellulose-bound total protein, stained using REVERT™ total protein staining kit (926-1101, LI-COR™) and analysed at 700 nm with the Odyssey FC image analyser, unless otherwise stated.

### 2.4. siRNA-Mediated Knockdown

Cells (2 × 10^4^) were cultured in CM in 24-well plates for 24 h and transfected with Dicer-substrate siRNAs using Lipofectamine™ RNAiMAX for 48 h as described by the manufacturer (Invitrogen, 13,778,030) using 30 nM C-siRNA1, C-siRNA2, C-siRNA3 or S. siRNA (Integrated DNA Technologies, BVBA, Leuven, Belgium, #73584944, #7358947, #7358950, #51-01-19-09).

### 2.5. Confocal Microscopy

Cells were cultured on a sterile 16 mm^2^ microscope coverslip, washed with ice-cold phosphate-buffered saline (PBS, Lonza, BE17-516F) and fixed with ice-cold 4% (*w*/*v*) paraformaldehyde for 10 min at room temperature. Fixed cells were permeabilised with 0.1% (*v*/*v*) Triton™ X-100 (Merck, T8787) in PBS for 10 min at room temperature, then washed in PBS for 15 min. Permeabilised cells were incubated for 60 min at room temperature in blocking buffer (PBS, 0.2 M glycine, 10% *v*/*v* foetal calf serum). Cells were incubated overnight at 4 °C with α-collagen III (ab7778, Abcam, UK) primary antibody. Cells were washed in PBS for 40 min at room temperature, then incubated with Goat α-Rabbit Alexa Fluor^®^ 594 secondary antibody (ab150080, Abcam, UK) for 1.5 h at room temperature. Cells were washed in PBS for 1 h at room temperature, then counterstained with 300 nM 4′,6-diamidino-2-phenylindole (DAPI)-hydrochloride (D1306, Thermo Fisher Scientific) for 1 min at room temperature. Cells were washed in PBS for 1 min at room temperature, then mounted with Fluorosave™ (345,789, Millipore). For phalloidin staining, fixed and permeabilised cells were incubated at room temperature for 1 h with 165 nM Alexa Flour™ 488 Phalloidin (A12379, Invitrogen) in PBS, then washed and stained with DAPI as described above. Images were captured and processed with an LSM 800 confocal microscope with a 63 Å~/1.4 NA oil immersion objective using ZEN 2.3 (blue edition) software (Carl Zeiss GmbH, Jena, Germany). ImageJ (1.52 k) was used to measure the fluorescence of target fluorophore-conjugated secondary antibody after subtracting background. Fluorescence intensity was calculated by dividing the fluorescence mean values of the selected region of interest (ROI) by area in square micrometres (μm^2^) of the selected ROI. The intensity levels were semi-quantified by calculating the fluorescence intensity mean value in the test cells relative to the fluorescence intensity of the control cells.

### 2.6. Construction of gRNA Targeting Vectors and Identification of Genome-Edited Cells

The pMD plasmid DNA targeting vector was derived from the gRNA cloning vector (gift from George Church Addgene ID 41824) [18]. A CRISPR target for murine *Col*3a1 exon 2 was identified using a design tool (http://crispr.mit.edu), two partially overlapping oligonucleotides designated pMD gRNAF and pMD gRNAR were synthesised (Integrated DNA technologies, BVPA, Leuven, Belgium) and 0.5 µM of each was annealed and extended in 1X Phusion^®^ HR buffer, 200 µM dNTPs and 1 unit Phusion^®^ DNA polymerase (New England Biolabs, M0530) in 50 µL, at 98 °C for 30 s, then 40 cycles at 98 °C for 10 s, 52 °C for 30 s and 72 °C for 30 s, followed by 72 °C for 2 min in a thermal cycler (MJ Scientific). Annealed DNA was purified by Nucleospin^®^ Gel and PCR clean-up (Machary-Nagal) and inserted into BspTI (Thermo Fisher Scientific., ER0831) linearised gRNA plasmid DNA by Gibson assembly according to the manufacturer’s instructions (New England Biolabs, E5510). NEB 5-alpha high-efficiency competent cells (New England Biolabs, E5510) were transformed with recombinant DNA. pMD recombinant plasmid DNA was prepared from bacterial cultures by affinity chromatography as described by the manufacturer (Nucleobond^®^ PC500EF columns, Machery-Nagal GmbH, Germany) and partially sequenced using the LKO.1 primer (Eurofins Genomics, Constance, Germany).

Heteroduplex screening analysis of genome-edited single-cell-derived colonies was performed using 40 ng of QuickExtract-isolated DNA (Lucigen, Cambridge, UK, QE09050), which was amplified with 300 nM of Col3a1Alt-RF and Col3a1Alt-RR primers with DreamTaq Green PCR master mix (ThermoScientific, K1081). Heteroduplex analysis of amplified DNA was performed using the Alt-R Genome Editing Detection Kit as described by the manufacturer (IDT, 1075932). Cell mutant alleles were identified by sub-cloning the same PCR-amplified DNA into the pJET1.2 vector using the CloneJET PCR cloning kit (Thermo Fisher Scientific, K1231), transformation into NEB 5-alpha competent cells, colony PCR with pJET1.2 specific primers (Thermo Fisher Scientific, K1231), DNA fragment purification (Nucleospin^®^ Gel and PCR clean-up, Machary-Nagal) and sequencing (Eurofins Genomics, Constance, DE) with the pJET1.2 Forward primer, all as described previously [1].

### 2.7. Oligonucleotides Primers

The sequences of oligonucleotides for murine *aP*2, *C*/*ebp*α, P*par*γ_2_, β-actin, *Col*1a1, *Col*4a1, *Col*6a1, *Fn*1 and *18*S rRNA used in this study have been described before [1]. The sequence of other oligonucleotides, synthesised by Integrated DNA Technologies, are shown in the table below:
pMDgRNAF: TTTCTTGGCTTTATATATC TTGTGGAAAGGACGAAACACCGACAG ATTATGTCATCGCAApMD gRNAR: GACTAGCCTTATTTTAA CTTGCTATTTCTAGCTCTAAAACTTG CGATGACATAATCTGGACol3a1Alt-RF: GGAAGAGGTTTATACTG CCAAGACol3a1Alt-RR: ACCCAATCTCCTGCTTT CTGCol3a1F: CTGTAACATGGAAACTGGG GAAACol3a1R: CCATAGCTGAACTGAAAA CCACC

### 2.8. Statistical Analysis

Statistical significance was determined from the mean +/- standard error of the mean (SEM) of a minimum of three independent experiments using the unpaired two-tailed Student’s *t*-test, Welch’s *t*-test, Mann–Whitney test, one-way analysis of variance (ANOVA) or two-way ANOVA, using GraphPad PRISM® 8.4.0 software. *p*-values ≤ 0.05 were considered significant.

## 3. Results

### 3.1. Col3a1 KD Suppresses 3T3-L1 Cell Differentiation

**Figure 1:**
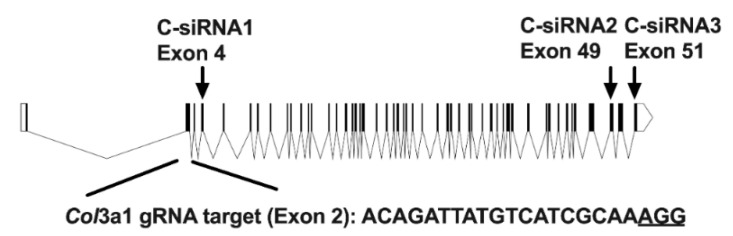
Schematic representation of the murine *Col*3a1 gene showing coding exons as shaded boxes. Coding exons 4, 49 and 51 are highlighted by arrows. The approximate target regions of the Dicer substrate small interfering RNAs (DsiRNAs) C-siRNA1, -2 and -3 are illustrated. The approximate location of the target sequence of the pMD plasmid vector guide RNA (*Col*3a1 gRNA) and protospacer adjacent motif (underlined) in coding exon 2 is also shown.

**Figure 2:**
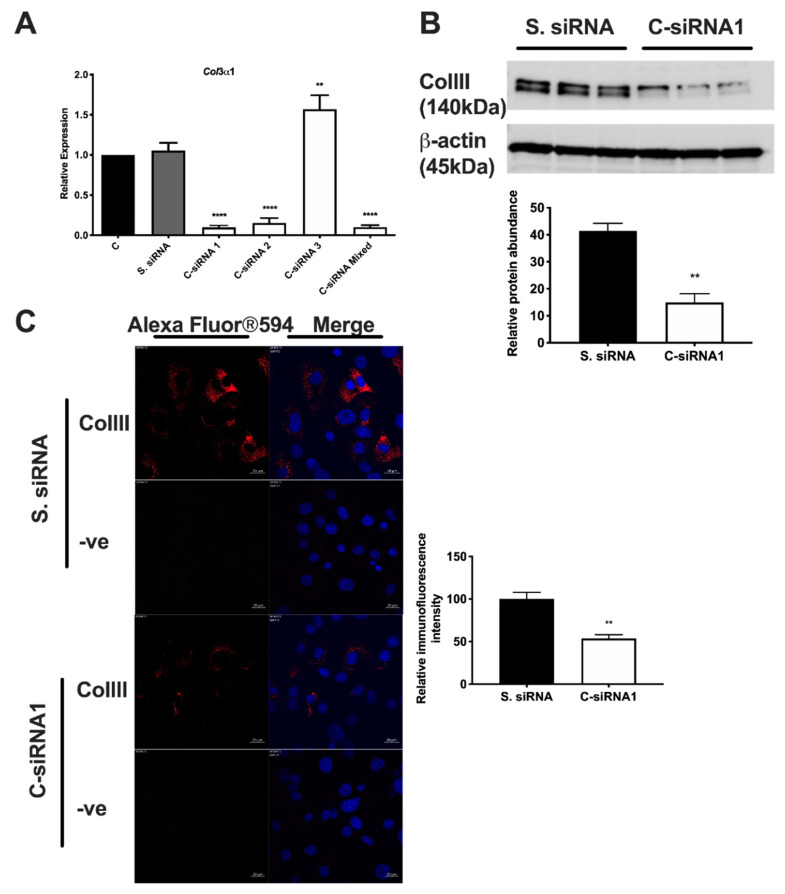
*Col*3a1 knockdown in 3T3-L1 cells. (**A**) Reverse transcription quantitative real-time PCR (RT-QPCR) analysis of *Col*3a1 gene expression in 3T3-L1 cells treated for 48 h with control scrambled S. siRNA, C-siRNA1, C-siRNA2, C-siRNA3 or C-siRNA1, -2 and -3 (C-siRNA Mixed). Histograms show the mean gene expression of three independent experiments, performed in triplicate, normalised to 18S rRNA relative to normalised *Col*3a1 gene expression in untreated 3T3-L1 cells (C) as the calibrator. Error bars show the standard error of the mean, n = 3. Statistical analysis shows one-way ANOVA and Dunnett’s multiple comparisons test of control C versus treated cells: ** *p* ≤ 0.01, **** *p* ≤ 0.0001. (**B**) Western blot analysis of ColIII (B-10) and β-actin proteins in 3T3-L1 cells treated with either control scrambled S. siRNA or *Col*3a1-specific C-siRNA1. Results show three independent experiments of cells treated with each siRNA. The molecular weight of each protein is indicated in kDa. The bar graph below shows a quantitative analysis of Western blots, showing the mean intensity of ColIII in S. siRNA (black bars) and C-siRNA1 (white bars) treated 3T3-L1 cells normalised to β-actin. Error bars are the standard error of the mean of 3 independent experiments (n = 3). Statistical analysis shows an unpaired two-tailed Student’s *t*-test of S. siRNA- versus C-siRNA1-treated cells, ** *p* ≤ 0.01. (**C**) Confocal images of α-ColIII (red) immunostained 3T3-L1 cells treated with the indicated siRNAs. Merged images show both ColIII and DAPI (blue) stained cells. Control images (-ve) show cells stained with the secondary antibody Alexa Fluor®594 and DAPI only. White size bar is 20 µm. The bar graph to the right is a quantitative analysis of confocal images of α-ColIII-stained 3T3-L1 cells treated with the indicated siRNAs using ImageJ software. The mean value of the fluorescence intensity of C-siRNA1 (white bars) treated cells relative to the control S. siRNA (black bars) treated cells is shown. Error bars are the standard error of the mean, n = 6. Statistical analysis shows a Mann–Whitney test of the fluorescence intensity of S. siRNA versus C-siRNA1-treated cells, ** *p* ≤ 0.01.

**Figure 3:**
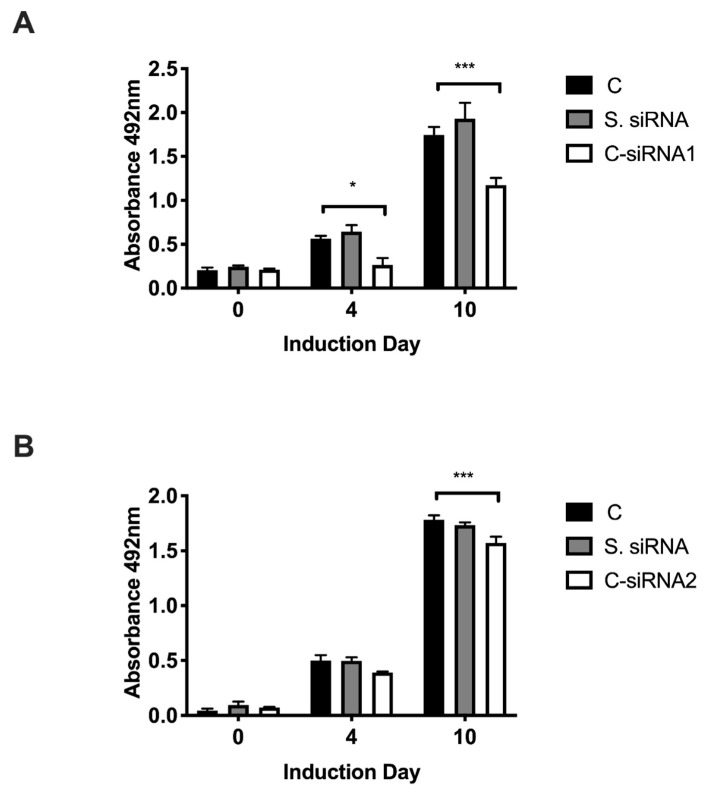
Semi-quantitative analysis of oil red O-stained 3T3-L1 cells at the indicated days of differentiation, either untreated (C) or treated with scrambled control S. siRNA, C-siRNA1 (**A**) or C-siRNA2 (**B**). Histogram is the mean of three independent experiments, performed in triplicate, and the error bars are the standard error of the mean. Statistical analysis shows the two-way ANOVA and Dunnett’s multiple comparisons test of untreated (C) versus C-siRNA1- or C-siRNA2-treated cells on the same day; * *p* ≤ 0.05, *** *p* ≤ 0.001.

We have previously shown that Gpr56 is required for adipocyte differentiation in 3T3-L1 cells [1], and so we investigated if one of its ligands, type III collagen (ColIII) encoded by the *Col*3a1 gene, is also involved in this developmental programme. *Col*3a1 gene knockdown (KD) was undertaken to investigate its role in the adipogenesis of 3T3-L1 preadipocytes. Initially, *Col*3a1 gene KD was optimised in 3T3-L1 cells transfected with three distinct Dicer substrate small interfering RNAs (DsiRNAs: C-siRNA1, -2 and -3), which target *Col*3a1 exons 4, 49 and 51 (Figure 1), as described in the Materials and Methods section. The abundance of *Col*3a1 gene transcripts and ColIII protein were monitored for 48 h post-transfection by reverse transcription quantitative real-time PCR (RT-QPCR), immunoblotting and immunostaining (Materials and Methods). RT-QPCR results show that C-siRNA1 and C-siRNA2, targeting sequences derived from Col3a1 exons 4 and 49 (Figure 1), were the most effective, reducing gene transcripts by 90% and 85%, respectively, versus untreated control 3T3-L1 cells (Figure 2A; C-siRNA1, C-siRNA2). C-siRNA3 elevates *Col*3a1 gene transcripts (Figure 2A) and was not investigated further, whereas the control DsiRNAs had no effect on *Col*3a1 gene transcripts (Figure 2A, S. siRNA). Western blot (Figure 2B) and immuno-stain (Figure 2C) analysis with ColIII antibodies showed a 60% (Figure 2B) and 47% (Figure 2C) reduction in ColIII protein in C-siRNA1-treated versus control S. siRNA-treated 3T3-L1 cells, consistent with the RT-QPCR data. Adipocyte differentiation of C-siRNA-treated 3T3-L1 cells, assessed by oil red O staining of neutral cell lipids (Materials and Methods), was significantly reduced at days 4 (54%) and 10 (33%) (Figure 3A, C-siRNA1) and day 10 (12%) (Figure 3B, C-siRNA2) relative to control (C) untreated cells (Figure 3A,B and Figure S1A,B). There was no significant difference in lipid deposition in untreated versus control S. siRNA-treated 3T3-L1 cells (Figure 3A,B, S siRNA). These results showed that *Col*3a1 siRNAs significantly and selectively reduced gene transcripts and ColIII protein and that the resulting reduction in ColIII levels significantly impaired the ability of 3T3-L1 cells to execute the adipocyte development programme.

### 3.2. Col3a1^−/−^ Genome-Edited 3T3-L1 Cells

**Figure 4:**
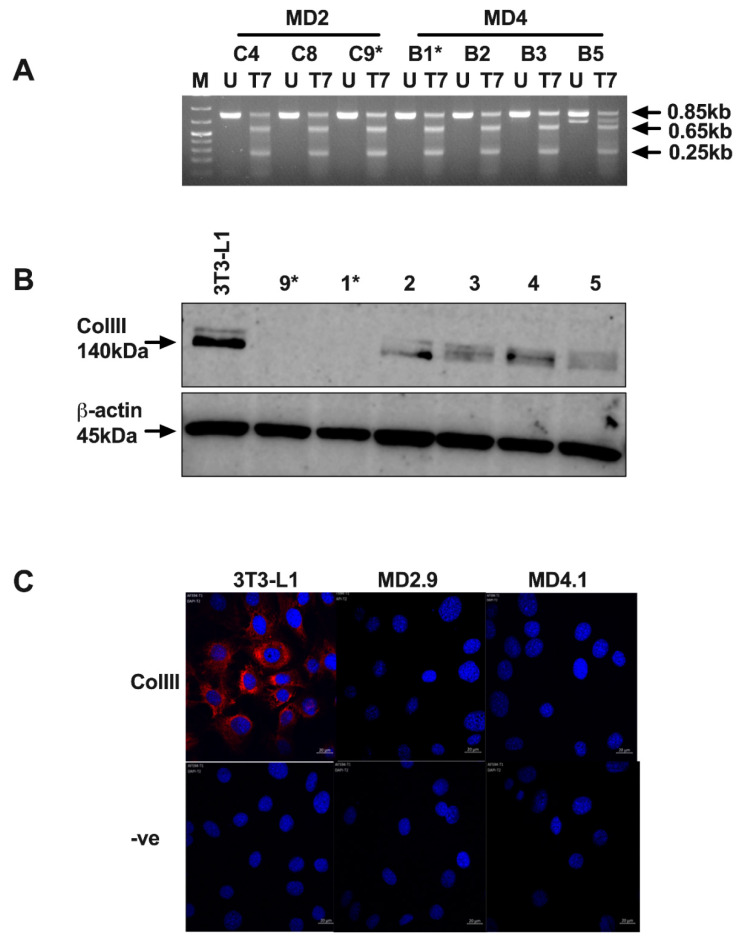
Identification and analysis of *Col*3a1 genome-edited 3T3-L1 cell clones. (**A**) Heteroduplex analysis of a selection of second-round single cell clones derived from first-round single cell clones designated MD2 or MD4. Agarose gel analysis of polymerase chain reaction-amplified DNA from a sample of MD2- or MD4-derived clones are shown, indicating the expected 0.85 kb fragment in each case in the untreated lanes (U) and the 0.65 kb and 0.25 kb fragments that show the formation of heteroduplexes and hence the presence of insertion/deletion mutations (INDELS), in enzyme-digested lanes (T7). M indicates the marker. Asterisks indicate the same clones marked by asterisks in (**B**). (**B**) Western blot analysis of parental 3T3-L1 cells and genome-edited cell clones (indicated by number) with α-ColIII (B-10) or α-β-actin, with arrows indicating the expected 140 kDa and 45 kDa proteins. Asterisks indicate clones selected for further study. (**C**) Confocal images of α-ColIII immunostained (ColIII, red) and DAPI-stained (blue) parental 3T3-L1 and genome-edited MD2.9- and MD4.1-derived cell clones. Control images (-ve) show cells immunostained with secondary antibody and DAPI only. White size bar is 20 µm.

In order to confirm the *Col*3a1 KD results and investigate the role of ColIII in adipogenesis further, *Col*3a1^−/−^ 3T3-L1 cells, which lack a functional *Col*3a1 gene, were created by genome editing. A pMD plasmid DNA *Col*3a1 guide RNA (gRNA) targeting vector that is specific for sequences located in *Col*3a1 exon 2 (Figure 1) was designed and created for genome editing as described in the Materials and Methods. 3T3-L1 cells were transfected with pMD, CAS9 and pC1 plasmid DNA, transiently selected with puromycin and single-cell-derived clones isolated and cultured, all as described in the Materials and Methods. Two rounds of single cell cloning were undertaken, and mutation screening by heteroduplex analysis was performed to identify clones with insertion/deletion mutations (INDELS) in the *Col*3a1 gene (Materials and Methods). Results of the analysis of a selection of second-round MD2 and MD4-derived single cell clones are shown in Figure 4.

Heteroduplex analysis revealed the presence of INDEL mutations within the *Col*3a1 target region of all clones shown, as indicated by the appearance of 0.65 kb and 0.25 kb DNA fragments in bacteriophage T7 endonuclease (T7) digested DNA (Figure 4A). MD2- and MD4-derived single cell clones were selected for further investigation for ColIII protein production by Western blot analysis. Results demonstrated that ColIII protein was not detected in clones MD2.9 and MD4.1 (highlighted by * in Figure 4B), whereas it was readily detected but reduced in all other sub-clones shown, compared to the parental 3T3-L1 cells (Figure 4B). The complete loss of ColIII protein in MD2.9 and MD4.1 was confirmed by confocal analysis of cells immunostained with α-ColIII antibodies. Results showed readily detectable staining of ColIII in 3T3-L1 cells that was completely absent in MD2.9 and MD4.1 cells (Figure 4C). Together, these results confirmed that MD2.9 and MD4.1 single cell clones lack detectable ColIII protein.

**Figure 5:**
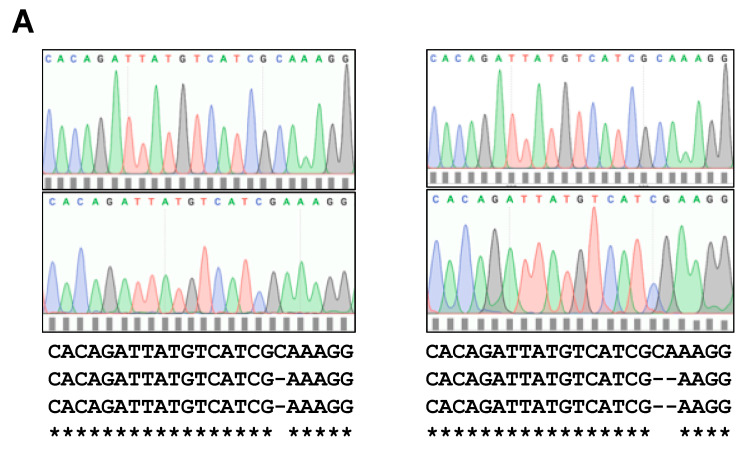

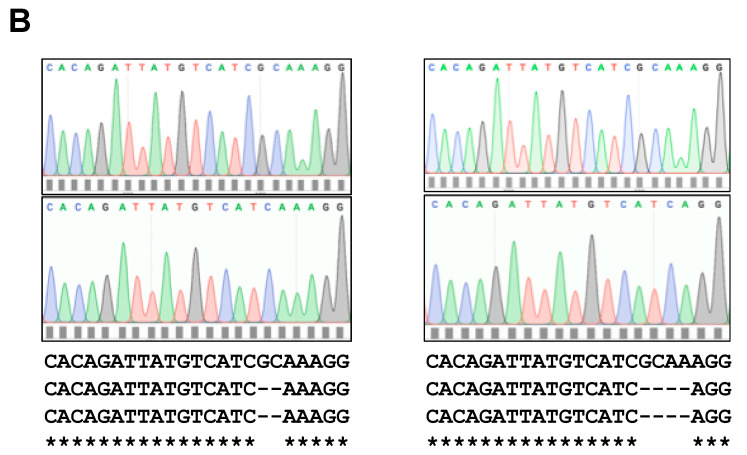
Partial nucleotide sequence of inactivating deletion mutations in *Col*3a1 genome-edited 3T3-L1 cells. Shown are the four-colour DNA chromatograms and corresponding nucleotide sequences of both *Col*3a1 alleles in MD2.9 (**A**) and MD4.1 (**B**) genome-edited single cell clones. Both normal and genome-edited cell clone sequences are shown. Regions of sequence identity are indicated by single-letter nucleotide code and are highlighted by asterisks. Deletion of nucleotides are shown by a -.

The presence of deletion mutations in both *Col*3a1 alleles of MD2.9 and MD4.1 cells was confirmed by DNA cloning and sequencing of PCR-amplified *Col*3a1 gene DNA fragments from MD2.9 and MD4.1 cell DNA (Materials and Methods). The results demonstrated that both MD2.9 and MD4.1 3T3-L1 cells had compound heterozygous frameshift deletion mutations in the gRNA target region, which abolished ColIII production. In MD2.9 cells, one allele had a single nucleotide deletion and one allele a two-nucleotide deletion (Figure 5A), whereas MD4.1 had alleles with two and four nucleotide deletions, respectively (Figure 5B). These results confirmed that MD2.9 and MD4.1 cells lack ColIII due to inactivating deletion mutations in both *Col*3a1 alleles in each case, and were therefore designated *Col*3a1^−/−^ 3T3-L1 cells.

### 3.3. Inactivation of Col3a1 Abolishes 3T3-L1 Cell Adipogenesis

**Figure 6:**
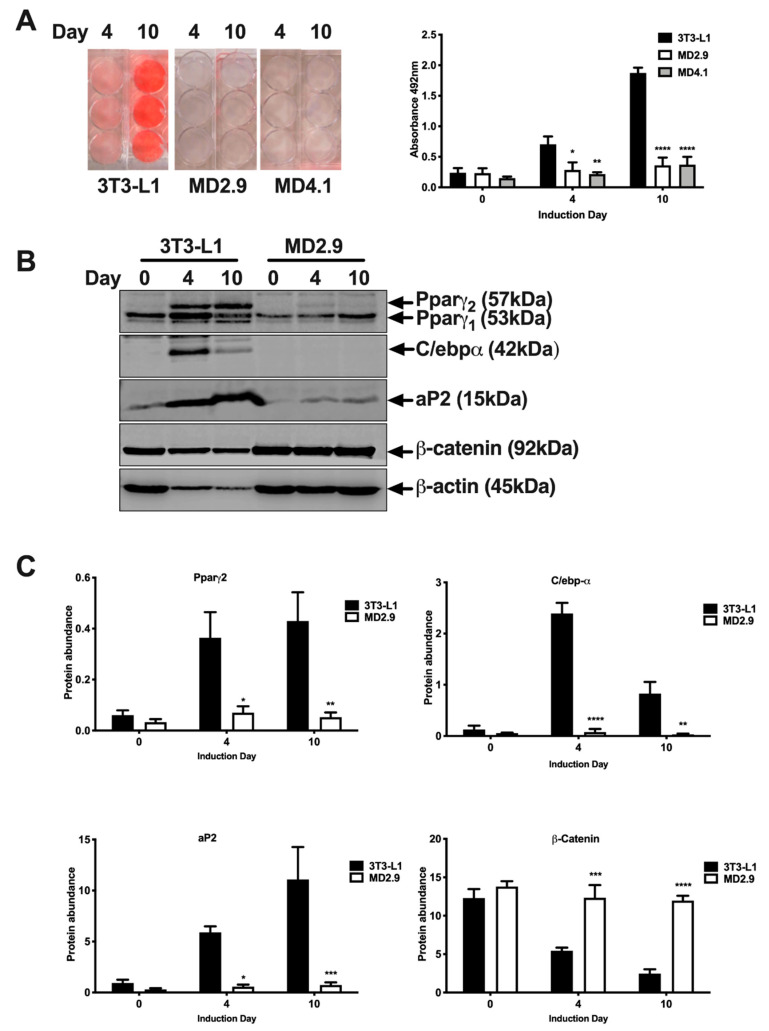
Differentiation of *Col*3a1^−/−^ 3T3-L1 cells. (**A**) Image and semi-quantitative analysis of Oil red O staining of the indicated cell lines at days of the adipocyte differentiation programme shown. Histogram shows the mean value of three independent experiments, performed in triplicate, and error bars the standard error of the mean (n = 3). Statistical analysis shows two-way ANOVA and Dunnett’s multiple comparisons test of 3T3-L1 versus *Col*3a1^−/−^ 3T3-L1 MD2.9 or MD4.1 cells for each day of induction; * *p* ≤ 0.05, ** *p* ≤ 0.01, **** *p* ≤ 0.0001. (**B**) Western blot analysis of the indicated cells at the days of adipocyte differentiation shown with Pparγ, C/ebpα, aP2, β-catenin or β-actin antibodies. Proteins of the expected size in kDa are indicated by arrows in each case. (**C**) Quantitation of Western blot analysis of the indicated proteins in the cells shown at differentiation induction days 0, 4 and 10. Histograms are the mean of three independent differentiation and Western blot experiments with each antibody and error bars show the standard error of the mean (n = 3). Statistical analysis shows two-way ANOVA and Sidak’s multiple comparisons test of 3T3-L1 versus *Col*3a1^−/−^ 3T3-L1 MD2.9 cells on each induction day; * *p* ≤ 0.05, ** *p* ≤ 0.01, *** *p* ≤ 0.001, **** *p* ≤ 0.0001.

Next, we investigated the differentiation of Col3a1^−/−^ 3T3-L1 cells. Adipocyte differentiation was investigated in *Col*3a1^−/−^ 3T3-L1 cells and compared with parental 3T3-L1 cells. Oil red O staining of neutral cell lipids and quantitation was performed at days 0, 4 and 10 of differentiation (Materials and Methods). Results showed that lipid deposition was completely abolished in MD2.9 and MD4.1 *Col*3a1^−/−^ 3T3-L1 cells (Figure 6A). Oil red O staining was dramatically reduced in either MD2.9 or MD4.1 versus 3T3-L1 cells at days 4 (60–70%) and 10 (80%) of the developmental programme. Furthermore, Western blot analysis showed a corresponding failure to induce regulators and markers of adipocyte differentiation in *Col*3a1^−/−^ 3T3-L1 cells (MD2.9) compared to 3T3-L1 cells (Figure 6B). Pparγ_2_, C/ebpα and aP2 were all significantly reduced compared to 3T3-L1 cells at days 4 (81%, 97% and 90%, respectively) and 10 (88%, 96% and 93%, respectively) (Figure 6C).

Next, we assessed levels of a known inhibitor of adipogenesis, β-catenin, by Western blot analysis in *Col*3a1^−/−^ 3T3-L1 cells. This analysis showed that *Col*3a1^−/−^ 3T3-L1 cells (MD2.9) had significantly elevated levels of active β-catenin at days 4 (2.3 fold increase) and 10 (4.9 fold increase) of differentiation versus 3T3-L1 cells (Figure 6B,C). β-catenin levels significantly declined in 3T3-L1 cells at day 4 (*p* ≤ 0.01) and day 10 (*p* ≤ 0.0001) of differentiation but not in *Col*3a1^−/−^ (MD2.9) cells (two-way ANOVA with Dunnett’s multiple comparisons test on day 4 and day 10 3T3-L1 or MD2.9 versus 3T3-L1 on day 0). Analysis also revealed an expected decline of β-actin protein levels with differentiation of 3T3-L1 cells (Figure 6B). However, this was not observed for β-actin in *Col*3a1^−/−^ 3T3-L1 cells (MD2.9), which is also consistent with the block in differentiation observed (Figure 6B). Together, these data confirmed that 3T3-L1 preadipocyte cells required ColIII in order to execute the adipogenesis developmental programme.

### 3.4. Col3a1 Cells Have Reduced Actin Stress Fibres

**Figure 7:**
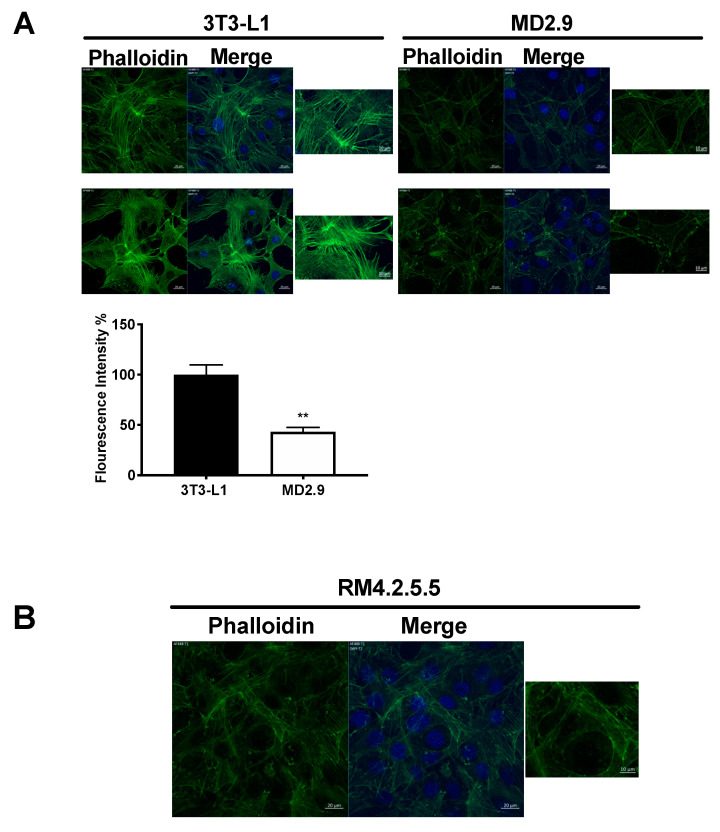
Confocal immunofluorescence analysis of actin stress fibres. (**A**) Confocal images of phalloidin (green) stained and DAPI (blue) stained (Merge) cell lines indicated. White size bars are 20 µm and 10 µm (adjacent magnified image). Below is a histogram of quantitative analysis of fluorescence intensity of phalloidin-stained cells using ImageJ software. The histogram shows the mean percentage of fluorescence intensity of *Col*3a1^−/−^ MD2.9 cells relative to control 3T3-L1 cells and error bars are the standard error of the mean (n = 6). Statistical analysis shows a Mann–Whitney test of *Col*3a1^−/−^ MD2.9 versus 3T3-L1 cells, ** *p* ≤ 0.001. (**B**) Confocal images of phalloidin (green) stained and DAPI (blue) stained (Merge) *Gpr*56^−/−^ RM4.2.5.5 cells. White size bars are 20 µm and 10 µm (adjacent magnified image).

Previously, we have shown that Gpr56^−/−^ 3T3-L1 cells that lack Gpr56 have reduced actin stress fibres [1], so we examined actin stress fibres in *Col*3a^−/−^ 3T3-L1 cells. Phalloidin staining was used to examine the actin cytoskeleton in both 3T3-L1 and *Col*3a1^−/−^ 3T3-L1 (MD2.9) cells (Materials and Methods). Results showed a change from abundant actin stress fibres in parental 3T3-L1 preadipocytes to significantly reduced stress fibres, reduced phalloidin staining intensity (67%) and increased cellular cortical staining in *Col*3a1^−/−^ 3T3-L1 (MD2.9) cells (Figure 7A). Similar results were also seen in *Gpr*56^−/−^ 3T3-L1 (RM4.2.5.5) cells (Figure 7B). These results showed there is a similarity in 3T3-L1 cells that lack either ColIII or Gpr56 with a significant reduction in overall phalloidin staining, a reduction in actin stress fibres and increased actin cortical staining.

### 3.5. Col3a1^−/−^ 3T3-L1 Cells Show Reduced Cellular Adhesion

**Figure 8:**
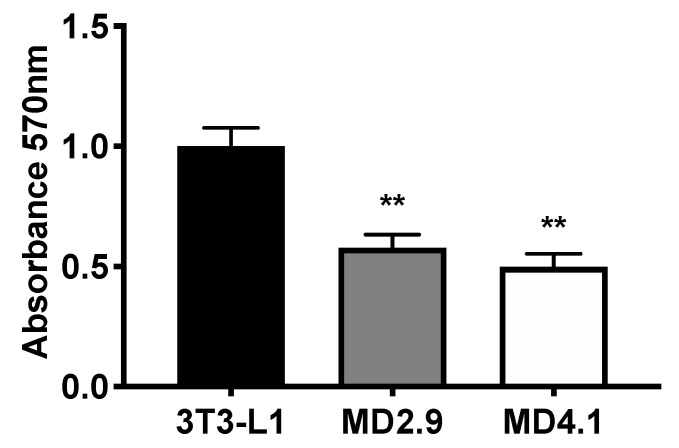
Adhesion of the indicated cell lines determined by quantitative analysis of crystal violet-stained cells relative to control 3T3-L1 cells. Statistical analysis shows one-way ANOVA with Dunnett’s multiple comparisons test of 3T3-L1 versus Col3a1^−/−^ 3T3-L1 MD2.9 or MD4.1 cells, ** *p* ≤ 0.01. Histograms are the mean of three independent experiments, performed in triplicate, and error bars are the standard error of the mean.

Actin stress fibres participate in cell adhesion. The reduction in actin stress fibres observed in *Col*3a1^−/−^ 3T3-L1 cells suggests this may have an impact on cellular adhesion. Therefore, we investigated adhesion of these cells. 3T3-L1 and *Col*3a1^−/−^ 3T3-L1 (MD2.9 and MD4.1) cells were plated in 96-well plates, incubated for 6 h at 37 °C and 5% CO_2_ then rinsed, fixed and stained with crystal violet, and the staining was quantified by measuring absorbance at OD 570 nm (Materials and Methods). Results showed significantly reduced staining in both MD2.9 and MD4.1 cells when compared to 3T3-L1 cells (Figure 8; MD2.9, 42%; MD4.1, 50%). These data showed that the loss of ColIII in 3T3-L1 cells reduced cellular adhesion, which is consistent with the reduction in actin stress fibres observed.

### 3.6. Col3a1^−/−^ 3T3-L1 Cells Show Changes in Extracellular Matrix Gene Expression

**Figure 9:**
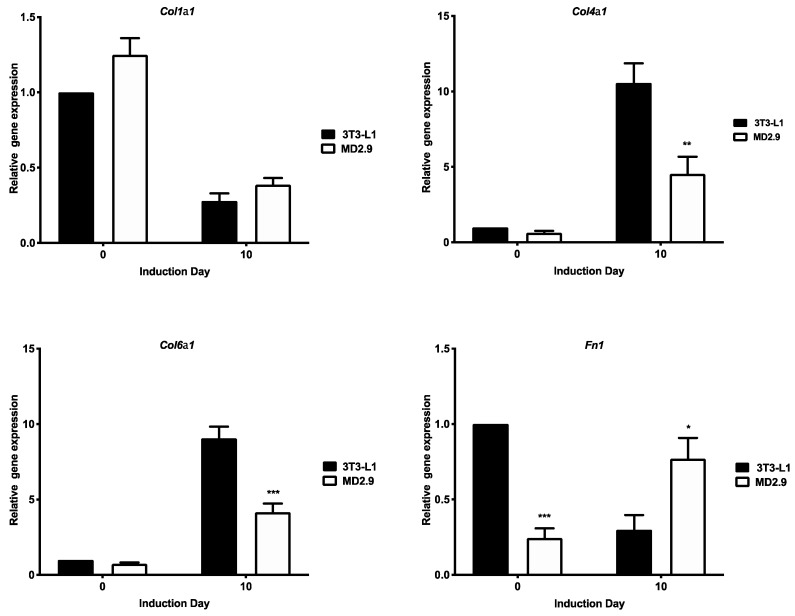
Analysis of selected extracellular matrix gene transcripts in *Col*3a1^−/−^ MD2.9 cells. The abundance of indicated gene transcripts in 3T3-L1 and MD2.9 cells are shown at days 0 and 10 of the adipogenic differentiation programme. Histograms show the mean values of three independent experiments, performed in triplicate, of each gene normalised to 18S rRNA relative to the normalised expression of the same gene at day 0 in 3T3-L1 cells, which were used as a calibrator. Error bars show the standard error of the mean (n = 3). Statistical analysis shows two-way ANOVA and Sidak’s multiple comparisons test of gene expression in 3T3-L1 versus MD2.9 cells on the same day; * *p* ≤ 0.05, ** *p* ≤ 0.01, *** *p* ≤ 0.001.

We have previously shown that loss of Gpr56 in 3T3-L1 cells results in changes in expression of gene transcripts encoding other ECM proteins in preadipocytes as well as following the adipocyte differentiation regime. Therefore, we investigated if loss of ColIII in 3T3-L1 cells also results in changes in abundance of ECM protein-encoding gene transcripts. RT-QPCR analysis of a selection of ECM-encoding gene transcripts in *Col*3a1^−/−^ 3T3-L1 (MD2.9) versus 3T3-L1 cells were examined at day 0 (preadipocytes) and day 10 of the differentiation programme. Results showed that the profile of *Col*1a1 transcripts were similar in both cell types at days 0 and 10 (Figure 9). Further analysis showed in both cases that *Col*1a1 transcripts are significantly repressed at day 10 compared to day 0 (two-way ANOVA with Dunnett’s multiple comparison test of day 10 versus day 0 for 3T3-L1, *p* ≤ 0.001). *Col*4a1 and *Col*6a1 transcripts were both significantly reduced at day 10 of differentiation in *Col*3a1^−/−^ 3T3-L1 (MD2.9) versus 3T3-L1 cells (Figure 9, 67% and 64%, respectively). Further analysis showed that *Col*4a1 and *Col*6a1 transcripts were both significantly induced at day 10 in 3T3-L1 cells only relative to day 0 levels (two-way ANOVA and Dunnett’s multiple comparison test for 3T3-L1 on day 10 versus day 0; *Col*4a1 *p* ≤ 0.0001, *Col*6a1 *p* ≤ 0.01), although there is also a trend for induction in *Col*3a1^−/−^ 3T3-L1 MD2.9 cells (Figure 9). *Fn*1 transcripts were significantly reduced at day 0 (75%) and induced at day 10 (2.5-fold) of differentiation in *Col*3a1^−/−^ 3T3-L1 (MD2.9) cells relative to 3T3-L1 cells on the same day (Figure 9). However, further analysis showed that *Fn*1 transcripts are repressed at day 10 relative to day 0 in 3T3-L1 cells only, whereas there is no statistically significant difference in abundance between 3T3-L1 day 0 and *Col*3a1^−/−^ 3T3-L1 (MD2.9) cells at day 10 (two-way ANOVA and Dunnett’s multiple comparison test for 3T3-L1 or *Col*3a1^−/−^ 3T3-L1 (MD2.9) on day 10 versus 3T3-L1 on day 0). These results showed changes in the profile of expression of *Col*4a1, *Col*6a1 and *Fn*1 extracellular matrix gene transcripts in *Col*3a1^−/−^ 3T3-L1 (MD2.9) preadipocytes as well as after 10 days’ induction regime treatment compared with 3T3-L1 cells, suggesting that other changes in the composition of extracellular matrix occur in the absence of ColIII.

## 4. Discussion

This study shows for the first time that type 3 collagen (ColIII) is required to execute the adipogenic development programme in 3T3-L1 preadipocyte cells. Partial KD of ColIII with siRNA significantly reduces lipid deposition during differentiation, whereas elimination of ColIII in genome-edited cells abolishes the differentiation process. Both *Col*3a1^−/−^ 3T3-L1 cell lines created by genome editing cannot produce mature ColIII due to compound heterozygous exon 2 deletion frameshift mutations of one and two nucleotides (MD2.9) and two and four nucleotides (MD4.1). MD2.9 and MD4.1 cells might express truncated ColIII proteins of 65/63 amino acids and 62/64 amino acids, respectively, which comprise signal peptide and partial von Willebrand factor type C domain regions only but cannot produce mature 1464 amino acid pre-protein polypeptides [19].

The phenotype of the *Col*3a1^−/−^ 3T3-L1 cells described in this study was very similar to the phenotype of *Gpr*56^−/−^ 3T3-L1 cells we have described previously [1]. The complete loss of either ColIII or Gpr56 both abrogate adipogenesis, whereas partial KD significantly reduces lipid deposition during adipogenesis. The loss of either protein in 3T3-L1 cells is also accompanied by (1) reduced phalloidin staining of F-actin; (2) reduced actin stress fibres; (3) reduced cell adhesion in preadipocytes; as well as (4) sustained β-catenin and (5) changes in ECM *Col*4a1, *Col*6a1 and *Fn*1 gene expression in both preadipocytes as well as in cells 10 days post-differentiation treatment. These observations suggest that a functional relationship exists between ColIII and Gpr56 in 3T3-L1 cells, which contributes to the successful execution of adipogenesis.

The reduction in actin stress fibres in the absence of either ColIII or Gpr56 in 3T3-L1 cells is intriguing and shows both molecules are required for their formation. This observation is completely consistent with previous studies, suggesting GPR56 mediates RHOA dependent F-actin accumulation and actin stress fibre formation [20]. Our results suggest that ColIII may bind Gpr56 to stimulate a signal transduction pathway, resulting in the formation of actin stress fibres in 3T3-L1 cells. Previous studies by others show COLIII binds GPR56 to stimulate RHOA activation via G-proteins Gα12/Gα13 in neuronal cells [2]. Furthermore, it is also established that RHOA activates ROCK1 and ROCK2 kinases, causing actin stress fibre formation [21]. The dramatic loss of actin stress fibres observed in 3T3-L1 preadipocytes lacking either ColIII or Gpr56 [1] strongly suggests that a similar signal transduction pathway involving ColIII, Gpr56, Gα12/Gα13, RhoA and Rock kinases operates in 3T3-L1 preadipocytes and is responsible for the organisation of these structures. The absence of ColIII is very likely a direct cause of actin stress fibre reorganisation in *Col*3a1^−/−^ 3T3-L1 cells where actin cytoskeleton stress fibres are disrupted and F-actin is reduced and relocalised. Adipogenesis requires actin cytoskeleton remodelling in order to proceed [22]. However, premature structural changes impair differentiation [23]. It is possible that premature cytoskeleton remodelling resulting from the loss of ColIII in preadipocytes described here also inhibits adipogenesis. Further studies are required to confirm this possibility.

Other mechanisms could contribute to the complete inhibition of adipogenesis observed in *Col*3a1^−/−^ 3T3-L1 cells in this study, including (1) reduced adhesion, (2) sustained active β-catenin and (3) altered ECM gene expression. Reduced cell adhesion is inhibitory to adipogenesis [24]. *Col*3a1^−/−^ 3T3-L1 cell adhesion is significantly reduced versus 3T3-L1 preadipocytes. ColIII provides an extracellular scaffold for cell attachment [10]. Cell adhesion is also reduced in fibroblasts with *COL*3A1 mutations [25] as well as in neurons of *Col*3a1^−/−^ mice [15]. ColIII interacts with cell adhesion-promoting molecules such as DDR1 [26] and integrins [27]. The sustained levels of active β-catenin in Col3a1^−/−^ 3T3-L1 cells suggest that the loss of adipogenesis might also be mediated by the anti-adipogenic influence of β-catenin. Wnt/β-catenin inhibits differentiation in 3T3-L1 by suppressing the expression of the adipogenic moderator molecules Ppary and C/ebpa [28]. ECM changes can both inhibit and enhance adipogenesis. In this study, the abundance of selected ECM gene transcripts changed in cells lacking ColIII. *Col*1a1 gene transcripts were similarly repressed in *Col*3a1^−/−^ 3T3-L1 and 3T3-L1 cells at day 10 versus day 0, which indicates that at least some of the changes normally accompanying adipogenesis still occur in the absence of ColIII. *Col*3a1^−/−^ 3T3-L1 cell *Col*4a1 transcripts were reduced compared with 3T3-L1 cells at day 10 and were only significantly induced in 3T3-L1 cells relative to day 0 levels. *Col*3a1^−/−^ 3T3-L1 cell *Col*6a1 transcripts were also reduced compared to 3T3-L1 cells and again were only significantly induced in 3T3-L1 cells at day 10. *Fn*1 transcripts were significantly reduced in *Col*3a1^−/−^ 3T3-L1 versus 3T3-L1 cells but were 2.5 times more abundant in the mutant cell line at day 10. However, only 3T3-L1 cell *Fn*1 transcripts significantly declined at day 10 versus 3T3-L1 day 0 cells, which suggests differentiating *Col*3a1^−/−^ 3T3-L1 cells sustain high levels of fibronectin that are similar to 3T3-L1 preadipocytes. Both reduced *Col*6a1 and sustained *Fn*1 transcripts could contribute to the abrogation of differentiation in *Col*3a1^−/−^ 3T3-L1 cells, as type VI collagen KD inhibits 3T3-L1 cell differentiation [29] and fibronectin inhibits adipogenesis [30]. Excessive deposition of ECM components is also implicated in altered insulin sensitivity in adipose tissue [31] and in 3T3-L1 cells [32]. Therefore, it is possible that the changes in ECM described here, caused by the loss of ColIII, may result in increased insulin resistance. This possibility requires further investigation.

Both the role of ColIII and its relationship with Gpr56 in adipocyte differentiation and the formation of actin stress fibres warrant further investigation. Administering recombinant type III collagen or enforced *Col*3a1 gene expression in *Col*3a1^−/−^ cells may complement the phenotype observed in these cells. This would facilitate a systematic examination of the downstream signalling events to be elucidated. Such an investigation may be enhanced by the creation of 3T3-L1 ColIII and Gpr56 double null cells to enable the functional relationship between these proteins to be explored by a combination of reconstitution studies with recombinant proteins and/or enforced expression or KD of downstream signalling molecules.

## 5. Conclusions

These studies show the importance of ColIII in the adipogenesis of 3T3-L1 cells. Further studies, including in vivo studies, are required to investigate this observation further to determine if type III collagen or downstream signalling pathways are potential targets for therapeutic intervention in obesity. For example, it is not known if Col3a1^−/−^ mice [13] have defects in adipogenesis. Furthermore, it would be useful to investigate and compare the expression of *Col*3a1 in the fat depots of genetic and diet-induced animal models of obesity to further investigate the relationship between ColIII and adipogenesis.

The phenotype of *Col*3a1^−/−^ 3T3-L1 cells is strikingly similar to that seen in *Gpr*56^−/−^ 3T3-L1 cells [1], demonstrating that ColIII is required for adipogenesis and suggesting that a functional relationship exists with Gpr56 in preadipocytes. 3T3-L1 preadipocytes lacking ColIII have reduced actin stress fibres, reduced cell adhesion, sustained active β-catenin and changes in the profile of expression of ECM genes including *Col*4a1, *Col*6a1 and *Fn*1. Each of these changes are likely to contribute to the block in adipocyte differentiation observed.

## Data Availability

The data presented in this study are available on reasonable request from the corresponding author.

## Supplementary Materials

The following are available online at https://www.mdpi.com/2218-273X/11/2/156/s1: Figure S1A: Image of Oil red O stained 3T3-L1 cells, independently treated with either scrambled control S. siRNA or C-siRNA1, at the indicated days of differentiation. Figure S1B: Image of Oil red O stained 3T3-L1 cells, independently treated with either scrambled control S. siRNA or C-siRNA2, at the indicated days of differentiation.
