# Supplementary material for: Type III Collagen is Required for Adipogenesis and Actin Stress Fibre Formation in 3T3-L1 Preadipocytes
Source: Biomolecules. 2021 Jan 25;11(2):156. doi: 10.3390/biom11020156 (PMC7911635; doi:10.3390/biom11020156)
Supplement: Supplementary file 1 [file biomolecules-11-00156-s001.pdf]

biomolecules

Article

Type III Collagen is Required for Adipogenesis and Actin Stress Fibre Formation in 3T3-L1 Preadipocytes.

Mohammad Al Hasan [1], Patricia E Martin [1], Xinhua Shu [1,2], Steven Patterson [1] and Chris Bartholomew [1,*]

Citation: Al Hasan, M..; Martin, P.E.; Shu, X.; Patterson, S.; Bartholomew, C. Type III Collagen is Required for Adipogenesis and Actin Stress Fibre Formation in 3T3-L1 Preadipocytes. *Biomolecules* **2021**, *11*, 156. https://doi.org/10.3390/biom11020156

Academic Editor: Carole Aimé
Received: 09 December 2020
Accepted: 20 January 2021
Published: 25 January 2021

Publisher's Note: MDPI stays neutral with regard to jurisdictional claims in published maps and institutional affiliations.

Copyright: © 2021 by the authors. Submitted for possible open access publication under the terms and conditions of the Creative Commons Attribution (CC BY) license (http://creativecommons.org/licenses/by/4.0/).

[1] Department of Biological & Biomedical Sciences, School of Health & Life Sciences, Glasgow Caledonian University, City Campus, Cowcaddens Road, Glasgow, G4 OBA, Scotland; Mohammad.al.hasan@outlook.com (M.A.H.); patricia.martin@gcu.ac.uk (P.E.M.); xinhua.shu@gcu.ac.uk (X.S.); steven.patterson@gcu.ac.uk (S.P.)
[2] Department of Vision Science, School of Health & Life Sciences, City Campus, Glasgow Caledonian University, Cowcaddens Road, Glasgow G4 OBA, UK
* Correspondence: c.bartholomew@gcu.ac.uk; Tel.: +44-0141-331-3213

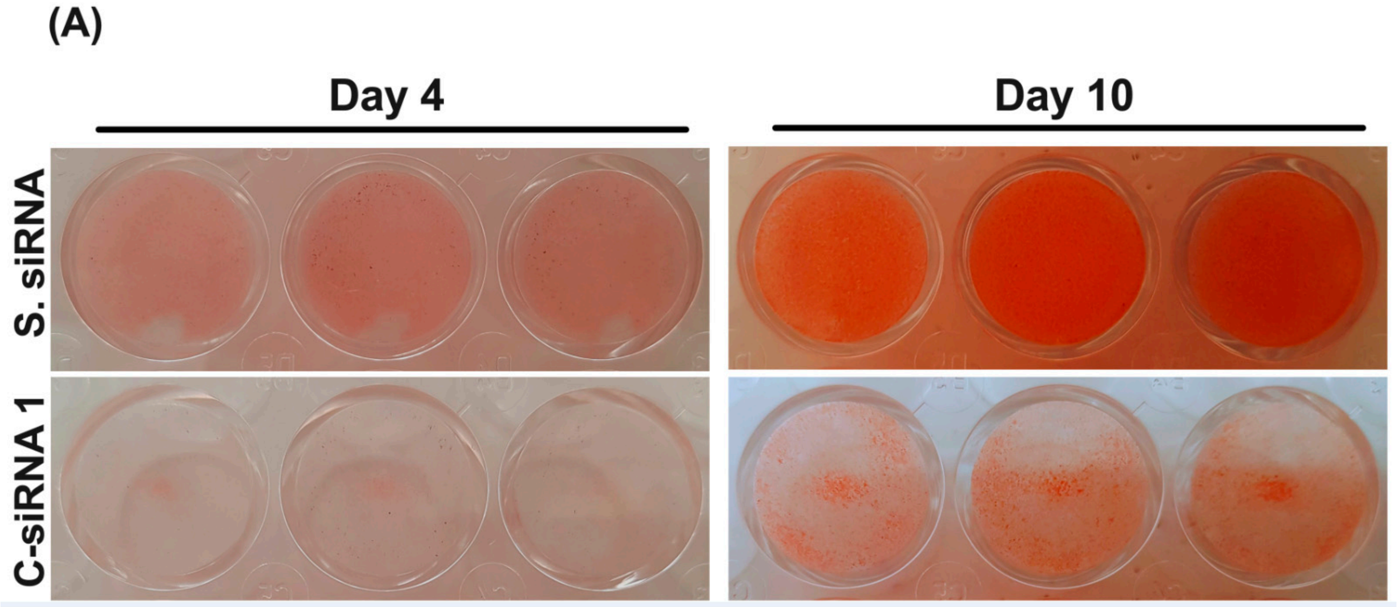

Figure S1. A: Image of Oil red O stained 3T3-L1 cells, independently treated with either scrambled control S. siRNA or C-siRNA1, at the indicated days of differentiation.

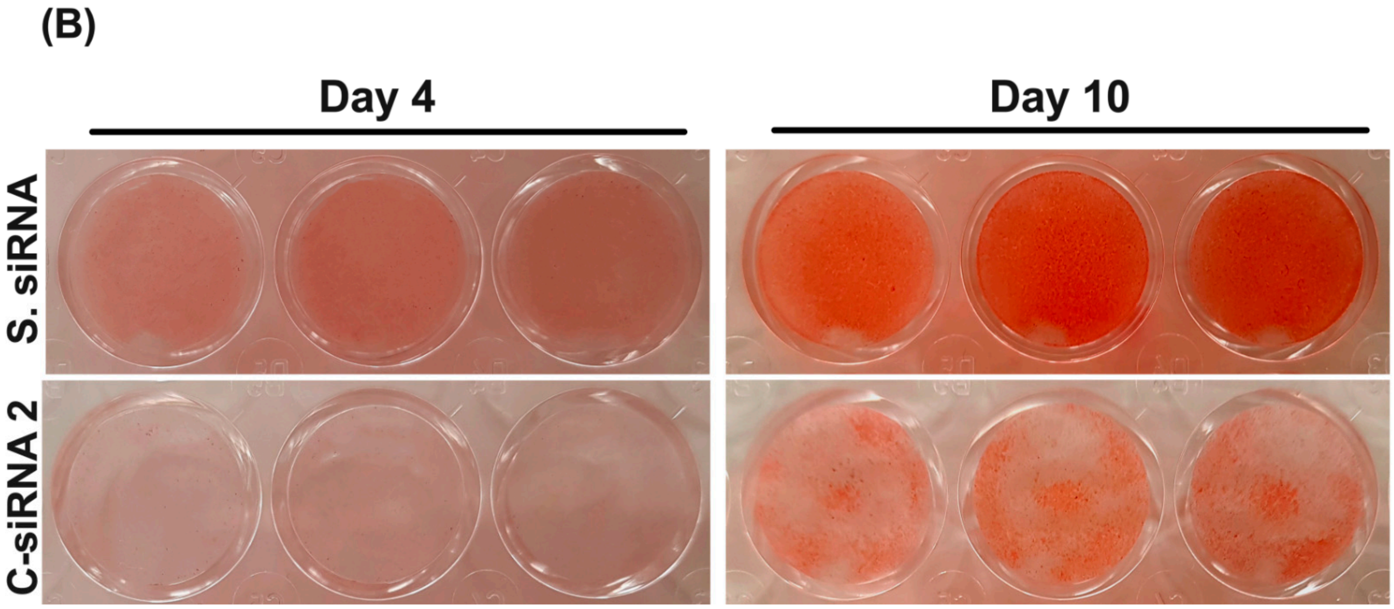

Figure S1. B: Image of Oil red O stained 3T3-L1 cells, independently treated with either scrambled control S. siRNA or C-siRNA2, at the indicated days of differentiation.
